# Effects of Specific Electric Field Stimulation on the Release and Activity of Secreted Acid Phosphatases from *Leishmania tarentolae* and Implications for Therapy

**DOI:** 10.3390/pathogens7040077

**Published:** 2018-09-27

**Authors:** Benjamin M. Dorsey, Cynthia L. Cass, David L. Cedeño, Ricardo Vallejo, Marjorie A. Jones

**Affiliations:** 1 Department of Chemistry, Illinois State University, Normal, IL 61790-4160, USA; bmdors2@gmail.com; 2 Millennium Pain Center, Bloomington, IL 61704-0303, USA; CCass@millenniumpaincenter.com (C.L.C.); dcedeno@millenniumpaincenter.com (D.L.C.); RVallejo@millenniumpaincenter.com (R.V.)

**Keywords:** *Leishmania*, leishmaniasis, secreted acid phosphatase, electric field, kinetic constants, glycosidase, potential therapy

## Abstract

Leishmaniasis is a neglected tropical disease with 1.6 million new cases reported each year. However, there are few safe, effective, and affordable treatments provided to those affected by this disease. Still under-appreciated as potential pharmaceutical targets, especially for cutaneous leishmaniasis infections, are the two isozymes of secreted acid phosphatase (SAP). These enzymes are involved in the survival of the parasite in the sand fly vector, and in infecting host macrophages. While the application of electric or electromagnetic fields as a medicinal therapeutic is not new, the utility of electric field application for the treatment of leishmaniasis is under studied. Studies involving the effects of electric fields on the cell secretion of SAP or the activity of SAP that has been secreted prior to electrical stimulation have not yet been reported. This work is the first report on the effect of specific electric fields on the activity of *Leishmania* *tarentolae* secreted acid phosphatases and the modulation of this secretion from the cells. In addition, the kinetic constants for the enzyme isoforms were determined as a function of days in culture and removal of carbohydrate from the glycosylated enzymes, while using a glycosidase, was shown to affect these kinetic constants.

## 1. Introduction

### 1.1. Leishmaniasis and Established Treatments

Human leishmaniasis is a neglected tropical disease carried by sand fly vectors that can have both zoonotic and anthroponotic transmission patterns [1]. The disease affects populations in Asia, India, the Middle East, Africa, Central and South America, and southern Europe [2], and it is caused by any of 20 species of the parasitic protozoan genus *Leishmania*. Leishmaniasis presents clinically in three forms: cutaneous, visceral, and mucocutaneous, with cutaneous being the most prevalent form. Over 1.6 million new cases of leishmaniasis diseases are reported yearly [2]. Current therapeutic options include treatment with either pentavalent antimony salts, amphotericin B, liposomal amphotericin B, miltefosine, pentamidine, paromomycin, ketoconazole, itraconazole, or fluconazole [3,4]. Individual treatment costs range from $20 to $252 USD per day and they must be administered for 20 to 120 days or longer, depending on healing process duration [5]. While *Leishmania* diseases are becoming more prevalent, there are few cost-efficient and effective drug therapies. Hence, electric field stimulation, a non-drug treatment, should be included in the search for new and effective treatments against leishmaniasis.

### 1.2. L. tarentolae Lifecycle and Secreted Acid Phosphatases

*L. tarentolae*, a species that normally infects reptiles, serves as a model species for anti-leishmaniasis treatment development, as they are easy to grow in culture and they do not infect humans. Indeed, since *L. tarentolae* can infect macrophages *in vitro*, and they are sensitive to current treatment options, they have utility in assessing infectivity and treatment development for *L. major*, *L. amazonesis*, and other species that infect humans [6]. The pathogenesis of *Leishmania* changes during the parasite life cycle, cycling between the flagellated promastigote that exists in the gut of the sand fly into the amastigote form that is found in macrophages of the infected host [7].

To survive under such biochemically strenuous conditions, *Leishmania* secretes acid phosphatases (EC 3.1.3.2; SAPs). Vannier-Santos et al. [8] reported that the secreted acid phosphatases have important roles in parasite infectivity perhaps by enhancing the host-parasite interactions. There are at least two SAP genes in other species of *Leishmania* [9], namely, secreted acid phosphatase 1 (SAP1) and secreted acid phosphatase 2 (SAP2). Secreted acid phosphatases, along with proteases, are important virulence factors for *Leishmania*, especially in the human parasite, *L. major* [10,11,12]. The secreted acid phosphatases are reported to be different from the membrane-bound acid phosphatase of Leishmania [13]. These secreted enzymes promote both parasite survival in the sand fly and infectivity in the macrophage. The formation and evolution of the parasitophorous vacuole in host macrophages by dephosphorylating macrophage membrane proteins and preventing macrophage hydrogen peroxide production is also reported to be modulated by secreted acid phosphatases [10,14,15]. While the genome of the promastigote form of *L. tarentolae* has been sequenced [16], it is not clear which of the available acid phosphatase sequences are functionally active.

The Michaelis-Menten kinetic parameters of secreted acid phosphatases change as a function of the parasite culture age. SAP enzymes that are monitored during the stationary phase of an *in vitro* culture of *L. major* have a larger V_max_ and a smaller K_m_ when compared to SAP enzymes that are monitored during the logarithmic phase of culture [17]. These constants stated for *Leishmania* SAPs are also affected by the state of N- and O-linked carbohydrate moieties found on the SAP mature glycoprotein [18]. However, studies involving effects of electric fields on the cell secretion of SAP or the activity of SAP that has been secreted prior to electrical stimulation have not yet been reported.

### 1.3. Electric Fields and L. tarentolae

The application of electromagnetic fields with different parameters is well documented for stimulating bone growth, decreasing arthritis, palliatively treating both post-operative edema and pain, and treating major depressive disorder [19]. In addition, electric field applied to mice infected with *Pseudomonas aeruginosa* lung infections resulting in significantly inhibited bacterial grown [20]. Yet, little work has been reported on assessing the potential of electric fields as a treatment for protozoan parasitic infections. Therapeutic electricity applied to *L. major* lesions on infected mice killed all *L. major* promastigotes [21]; however, the authors did not report a mechanism for this observation. We were especially interested in the potential effects of applying electric fields on inducing or inhibiting the secretion of acid phosphatase from *Leishmania* cultures, since these enzymes have been implicated to have important roles in host infectivity [10,14,15].

Here, we report the presence of functional *L. tarentolae* SAPs, and the effects on these enzymes of applying electric fields to *L. tarentolae* promastigotes *in vitro* for the first time. We found that application of electric fields affected *L. tarentolae* promastigotes motility, clumping, viability, and the ability of *L. tarentolae* to secrete these acid phosphatases. We tested the role of N-linked carbohydrate that is found on *L. tarentolae* SAPs, and found that the removal of N-linked carbohydrate moieties on SAP enzymes affected the experimentally determined Michaelis-Menten kinetic constants. Furthermore, we suggest that, due to their reported important contributions to overall parasite survival, *Leishmania* secreted acid phosphatases are of interest as potential therapeutic targets for the treatment of leishmaniasis.

## 2. Materials and Methods

### 2.1. In Vitro L. tarentolae Culture and Cell Viability Determination by MTT Analysis

Axenic *L. tarentolae* (ATCC 30143) promastigote cells were cultured at room temperature under sterile conditions in brain heart infusion (BHI; 37.0 g/L), supplemented with hemin (10 µM), penicillin (10,000 U/mL), and streptomycin (10 mg/mL), in canted 25 cm^2^ flasks (Corning Inc., New York, NY, USA), following the method of Morgenthaler, et al. [22]. *L. tarentolae* promastigote cell viability was assessed by the 3-(4, 5-dimethylthiazol-2-yl)-2, 5-diphenyltetrazolium bromide (MTT) viability assay [23]. Absorbance at 595 nm was determined with an iMark microplate reader (BioRad Laboratories, Hercules, CA, USA). The BHI growth medium alone served as a blank with its value absorbance (A595 nm/Hr incubation with MTT reagent) mean ± standard deviation (n = 4).

### 2.2. Microscopy of L. tarentolae

*L. tarentolae* promastigote cell motility, shape, and clumping were monitored while using a Jenco International, Inc. (Portland, OR, USA) inverted compound microscope Model CP-2A1. Daily images were captured while using a Google Pixel cellular phone (model G-2PW4100) at 400X. 

### 2.3. L. tarentolae Acid Phosphatase Enzyme Preparation

Samples of *L. tarentolae* promastigotes from each stage of the growth curve (lag, log, stationary, and senescence) were collected (2 mL) and centrifuged (2000× *g*, 10 °C, 10 min). The supernatants were collected and the cell pellets were immediately resuspended in a volume of BHI medium equal to the volume of supernatant that was collected from that same sample. The samples were then stored on ice until they were used in acid phosphatase (secreted or cell associated) enzyme assays.

### 2.4. Secreted Acid Phosphatase Enzyme Assay and Kinetic Assays

SAP activity was evaluated using *para*-nitrophenyl phosphate (*p*NPP) as the substrate following the method of Mendez, et al. [12]. The secreted acid phosphatase assay consisted of 500 µL 0.5 M sodium acetate buffer pH 4.5, 100 µL substrate (5 mg *p*NPP/1 mL 0.5 M sodium acetate buffer pH 4.5; 1.75 mM *p*NPP final concentration), and 300 µL SAP enzyme preparation from the *L. tarentolae* promastigote cell supernatant for a total volume of 900 µL. Assays for alkaline phosphatase activity in the supernatant fraction that was involved use of Tris-Base buffer (0.5 M, pH 8.3) added to the reaction and used to make substrate, with all other conditions as indicated above. The assays were performed at room temperature (24–26 °C) in 1.5 mL polypropylene microfuge tubes for 23 h, under apparent first order time [12], in the dark. The reaction was stopped with addition of 100 µL of 10 M sodium hydroxide and samples were then vortexed. Product formation was measured by absorption spectroscopy at 405 nm. BHI medium was used to replace the enzyme source for spectrophotometric blanks. The amount of *para-*nitrophenolate product (µM/23 hr) was calculated while using Beer’s Law from the measured absorbance values at 405 nm/23 hr absorbance and the molar absorptivity coefficient (ε = 18,000 cm^−1^∙M^−1^, www.sigmaaldrich.com).

SAP enzyme kinetics were determined in sodium acetate buffer (0.5 M, pH 4.5) by using *p*NPP prepared in assay buffer over a final substrate concentration range of 2.0 to 4000.0 µM.

### 2.5. Secreted Acid Phosphatase Enzyme Kinetic Assays with and without Pre-incubation with a Glycosidase

The *L. tarentolae* log phase promastigote cell supernatant was collected via centrifugation (2000× *g*, 10 min, 10 °C), incubated with glycosidase PNGase F (10 µL of PNGase F with activity of 10 U/µL was added per 25 mL enzyme pool; Promega, Madison, WI, USA) for 24 h at room temperature, and then used in kinetic assays, as described above. Control supernatant was incubated under the same conditions, but without added PNGase F.

### 2.6. Testing the Effects of Electric Fields on L. tarentolae Acid Phosphatase Activity and Secretion of Acid Phosphatase

#### 2.6.1. Effect on Secreted Enzyme Activity (Method 1)

To determine the effect of electric fields on secreted SAP from log phase *L. tarentolae* promastigotes, the samples were centrifuged (2000× *g*, 10 min, 10 °C), and the supernatant was collected. The cell pellet was resuspended in a volume of BHI equal to the volume of supernatant collected. The supernatant or pellet resuspension was transferred (taking care to deliver a consistent amount of cell supernatant or resuspended pellet) to each well into a 24-well plate (Falcon, 1.5 mL per well). Cell supernatant or pellet was exposed to a pulsed electric field for 30 min using a concentric, bipolar electrode (Model # CBJFM75, FHC Neuro Micro Targeting^TM^ Worldwide), as outlined in Figure S1. Method 1 allows for the evaluation of the direct effect of electric field on previously secreted SAP enzymes (in supernatant) or cell associated acid phosphatase activity remaining in the pellet. Control supernatant or pellet were from the same cell culture but were not exposed to electric fields.

#### 2.6.2. Effect on Enzyme Secretion (Method 2)

Log phase *L. tarentolae* promastigotes were transferred from 25 cm^2^ canted flasks into 24-well plates (Falcon; 1.5 mL per well), taking care to deliver a consistent amount of cell supernatant or resuspended cells to each well (as shown in Figure S2), and subjected to electrical stimulation or no electrical stimulation, as described above. After 30 min, the cells were centrifuged and the supernatant and cell pellet collected, and was separately used as the enzyme source in the acid phosphatase assay. Cell viability was monitored by the MTT assay. Method 2 allows for the evaluation of the effect on cell secretion of SAP by the electric field.

**Figure 1:**
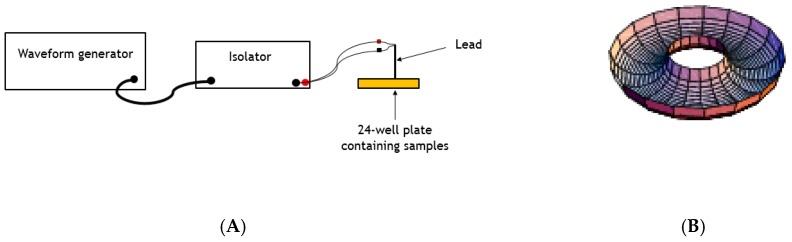
Simplified Model of the Experimental Setup and Electric Field. (**A**) Simplified model of the waveform generator, the signal isolator, and the concentric bipolar electrode shown. (**B**) The general shape of the electric field that was applied to either *L. tarentolae* whole cells or cell supernatant is shown.

Electrical pulses were produced by an arbitrary waveform generator (Siglent) that was coupled to signal isolators (World Precision Instruments, Sarasota, FL, USA) while using the following parameters: frequency of 50 Hz (400 µs pulse width) or 10,000 Hz (30 µs pulse width), current amplitudes of 100 µA, 150 µA, 200 µA, 250 µA, 300 µA, 400, or 500 µA, and the waveform polarity of cathodic monophasic, anodic monophasic, or symmetric biphasic pulses. A schematic diagram of the waveform generator, signal isolator, and electrical probe are shown in Figure 1A. Figure 1B shows the general shape of the electric field that was produced by the concentric bipolar electrode used in this study.

## 3. Statistical Analysis

Means for three replicates per group were statistically compared by two-tailed Student’s *t*-Tests with a 95% confidence level in Microsoft Excel.

## 4. Results

### 4.1. L. tarentolae Cell Viability with and without the Application of Electric Fields

**Figure 2:**
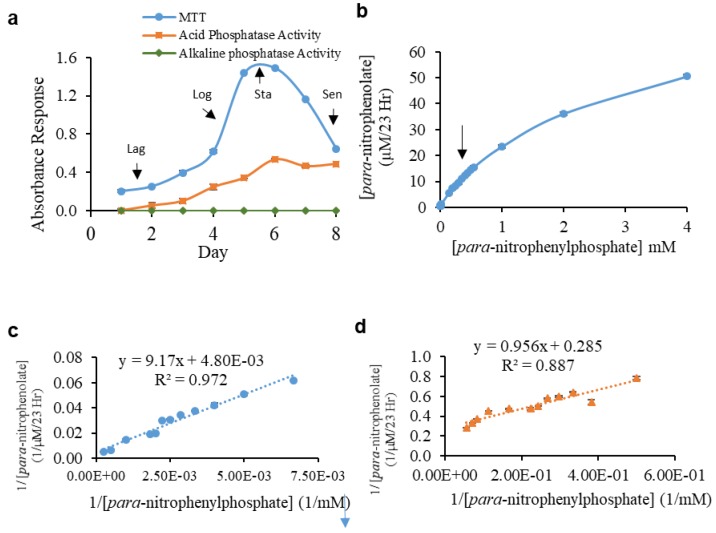
*L. tarentolae* Promastigote Secreted Acid Phosphatase (SAP) Secretion and Kinetic Parameters. (**a**) Cultured promastigote MTT activity (blue circles), and acid (red squares) or alkaline (green diamonds) phosphatase response over time (days in culture) are shown. Arrows indicate typical promastigote growth phases: Lag (days one to three), Log (days four to five), Stationary (Sta, days five to six), and Senescence (Sen, days seven to eight). (**b**) A typical velocity (*V*) vs. substrate concentration (S) curve utilizing *L. tarentolae* log phase promastigote supernatant as the enzyme source. The arrow marks the position within the curve with different slopes. (**c**,**d**) Lineweaver-Burk linear transformations using *L. tarentolae* log phase promastigote supernatant showing substrate production for either SAP1 (**c**) or SAP2 (**d**). Each point is the mean ± SD of three technical replicates.

**Figure 3:**
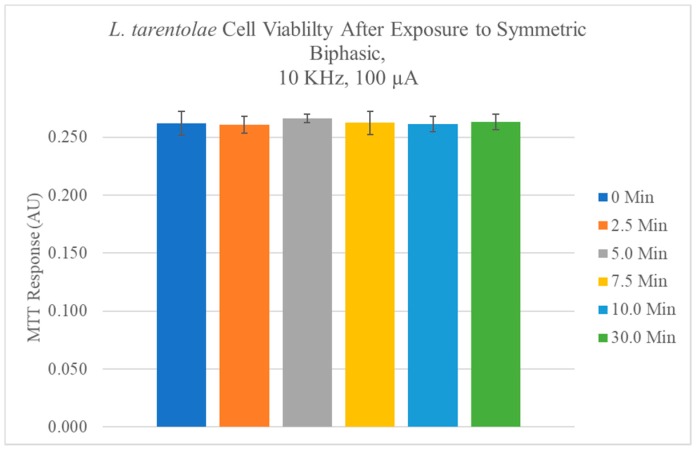
It shows the mean ± SD for n = 3 experiments in which cells were exposed to 10 KHz symmetrical biphasic electrical stimulation for various times then evaluated for cell viability using the MTT method. No significant differences relative to control cells with no electrical stimulation were observed.

A representative growth curve with lag, log, stationary, and senescence phases that were typical of cultured *L. tarentolae* promastigotes over the course of an eight-day culture period, determined by the MTT assay, is shown in Figure 2a. The growth curve was reproducible for promastigote cultures over the course of this study. No alkaline phosphatase activity was found in the supernatant fraction at any day in culture, whereas acid phosphatase was detected at each day in culture. Detectable secreted acid phosphatase activity tracked with the MTT response up to day six in culture (Figure 2a). However, after day six, as the MTT response decreased, there was no concomitant decrease in secreted acid phosphatase activity on days seven and eight (Figure 2a). The application of electric fields (50 Hz or 10,000 Hz, 100 to 500 µA, 30 min, monophasic cathodic, monophasic anodic, or symmetric biphasic waveforms) to *L. tarentolae* promastigote cells had no statistically significant effect on cell viability, as compared to control cells (Representative data are shown in Figure 3).

### 4.2. Microscopy of L. tarentolae with and without the Application of Electric Fields

**Figure 4:**
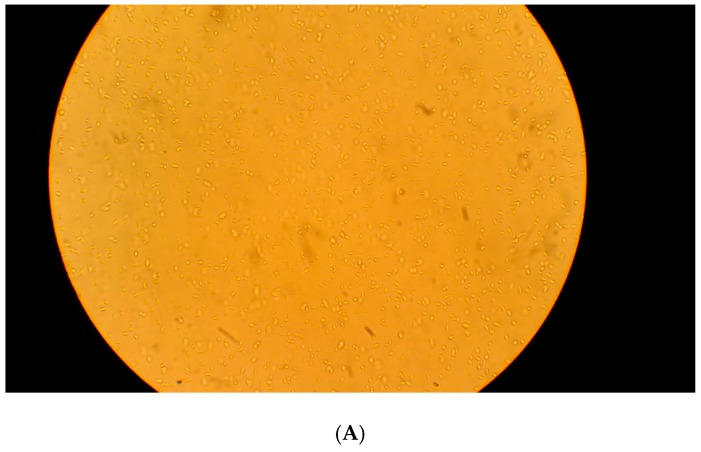

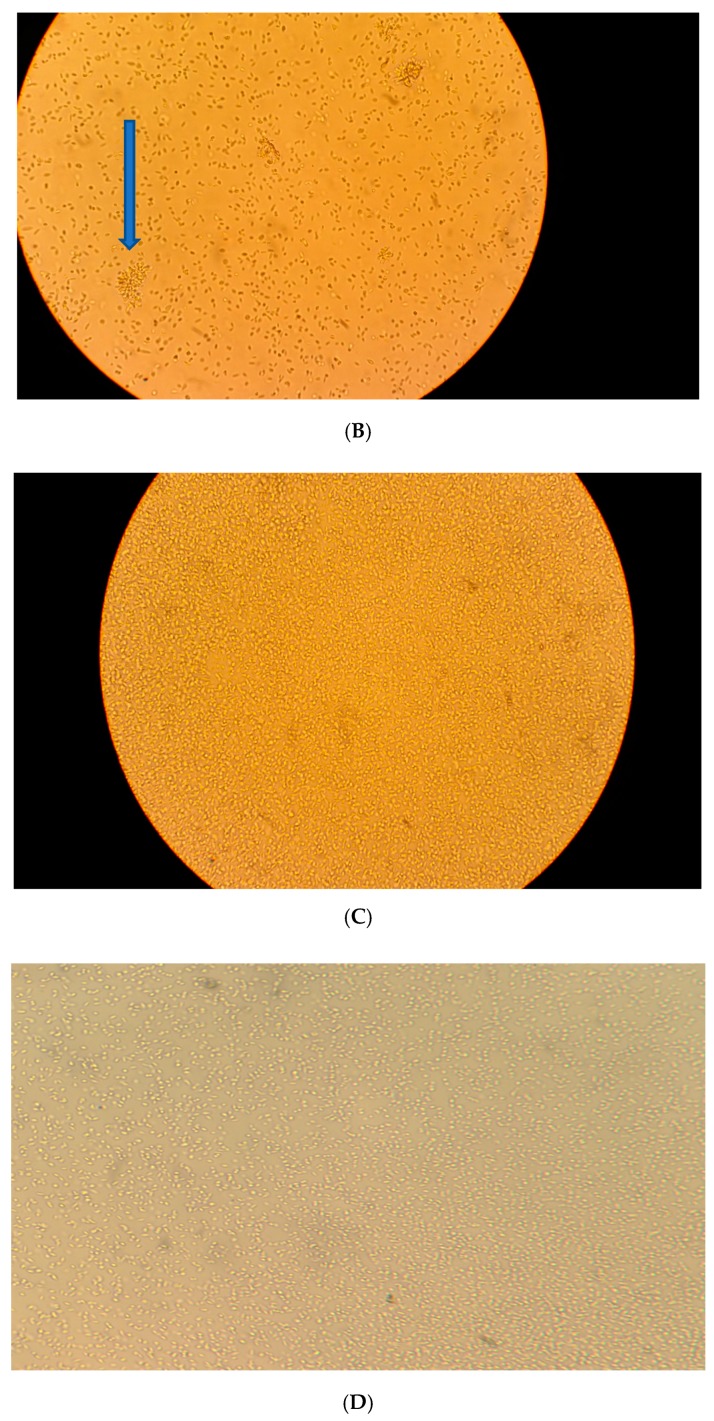

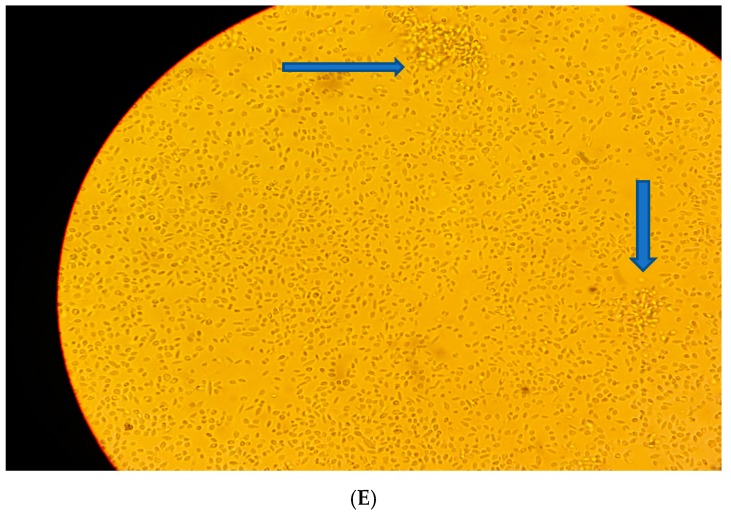
Phenotypes of Leishmania tarentolae promastigotes with or without electric stimulation. (**A**) *L. tarentolae* lag phase control cells (no electrical stimulation) viewed at 400× magnification. (**B**) *L. tarentolae* lag phase cells exposed to an electric field (50 Hz, 500 µA, 30 min, symmetric biphasic) viewed at 400× magnification. These cells exhibit several large clumps (arrow). (**C**) *L. tarentolae* lag phase cells exposed to an electric field (10,000 Hz, 500 µA, 30 min, symmetric biphasic) viewed at 400× magnification. These cells are highly packed into a single area. (**D**). *L. tarentolae* lag phase cells exposed to an electric field (50 Hz, 100 µA, 30 min, monophasic cathodic) viewed at 400× magnification. These cells appear more concentrated than typical *L. tarentolae* lag phase cells. (**E**) *L. tarentolae* lag phase cells exposed to an electric field (50 Hz, 100 µA, monophasic anodic) viewed at 400× magnification. The microscopic observations imply that the cells are moving toward the electrode during the electrode stimulation. These cells exhibit large clumps (arrows).

Log phase *L. tarentolae* promastigotes were observed by microscopy with and without the application of electric fields (Figure 4). Control cells that did not receive any electrical stimulation showed a standard, metacyclic phenotype (Figure 4A). Cells that were exposed to a symmetric biphasic electric field (50 Hz, 500 µA, 30 min) showed clumping (arrow in Figure 4B). After exposure to a symmetric biphasic electric field with higher frequency (10,000 Hz, 500 µA, 30 min), cells were packed into a single area (Figure 4C). After exposure to a monophasic cathodic electric field (50 Hz, 100 µA, 30 min), cells were more concentrated in one area, but few cell clumps were observed (Figure 4D). Similarly, cells that were exposed to a monophasic anodic electric field (50 Hz, 100 µA, 30 min) migrated toward the electrode during the stimulation (Figure 4E).

### 4.3. Enzyme Kinetics with and without Glycosidase Incubation

The *L. tarentolae* log phase supernatant secreted acid phosphatase appeared to exhibit Michaelis-Menten type enzyme behavior (velocity, V) as a response to increasing substrate (S) concentration (Figure 2B). However, a noticeable deviation from the apparent first order trend (shown by arrow in Figure 2B) occurred at 0.35 mM *p*NPP. Since the concentrations of SAP1 or SAP2 in the assay were unknown, comparisons of enzyme V_max_ after the different electrical treatments were performed while using the same enzyme source on the same day to control for the protein amounts.

The Lineweaver-Burk linear transformation of the V versus S curve, which typically produces a single straight line for a single, non-cooperative enzyme, produced two distinctively different straight lines for the *L. tarentolae* log phase supernatant SAP enzyme source. Figure 2C and two-dimensional (2D) (putative SAP1 and SAP2, respectively) show the Lineweaver-Burk linear transformations for the two distinct enzymes yielding different calculated kinetic constants. Putative SAP1 was present and detectable during the course of the entire eight-day growth curve (Table S1). The SAP1 K_m_ for substrate varied over a 1.7 fold range as a function of day in culture. However, the SAP1 V_max_ increased four-fold with culture age up to day five of the growth curve (Table S1), and remained at this level thereafter. Incubation of day eight supernatant with the PNGase F glycosidase, that removes N-linked carbohydrates, modestly changed the experimental K_m_ and V_max_ values of SAP1 relative to the untreated supernatant from the same day in culture. The K_m_ decreased by 24.4%, and the V_max_ increased by 13.5% (Table S1).

Putative SAP2 was not detectable during the first two days of the *L. tarentolae* promastigote growth curve, but it became detectable on day three, and remained detectable through day eight (Table S2). The K_m_ value for SAP2 remained in the same order of magnitude from day three to five of culture. The K_m_ values modestly increased from day six to eight, indicating that the enzyme became less efficient at binding substrate as the culture aged (Table S2). The V_max_ value of SAP2 remained consistent with culture age with moderate increases in the senescence phase (Table S2). Co-incubation with PNGase F glycosidase increased the K_m_ and V_max_ values of SAP2 relative to the untreated supernatant from the same day in culture by 1335% and 1507% for the K_m_ and V_max_, respectively (Table S2). Over the eight-day growth curve, both the V_max_ and K_m_ values for SAP 1 were substantially larger than for SAP2.

### 4.4. Effect of Electric Fields on Secreted and Pelleted Acid Phosphatase Activity (Method 1)

We examined the effect of applying electric fields directly to the enzyme source prior to its use in the acid phosphatase enzyme assay (Method 1, Figure S1). The application of a 50 Hz, monophasic cathodic pulsed electric field to *L. tarentolae* supernatant or pellet followed by the assessment of SAP activity resulted in modest measurable effects in both the supernatant (Table S1) and pellet (Table S1) activities that were significantly different from controls. A positive difference between the treatment and control means indicated that the applied electric field increased SAP activity, secretion, or secretion and activity. Conversely, a negative value indicated that the electric field treatment decreased SAP activity, secretion, or secretion and activity, relative to no treatment. The application of 100 µA pulses had the greatest effect on supernatant SAP activity (5% increase as compared to control, Table S1). The pellet activity was largely uninfluenced by this electric field (Table S1). No apparent trends were observed in the supernatant or pellet groups, under these conditions.

The application of 50 Hz, symmetric biphasic pulses, with amplitudes that ranged from 100 to 500 µA, to *L. tarentolae* supernatant or pellet followed by the assessment of SAP activity resulted in very modest measurable significant effects in both the supernatant and pellet activity from their controls (Table S1). The largest effect that was observed on supernatant samples was the 4% activation from the 150 µA pulse compared to control. The two largest effects observed on pellet samples were the 6% activation from the 100 µA pulse as compared to control, and the inhibition by 6% from the 200 µA pulse when compared to control, however, no consistent trend was observed (Table S1). No apparent trends were observed in the supernatant or pellet groups, under these conditions.

The application of a 50 Hz, monophasic anodic pulses, with amplitudes that ranged from 100 to 500 µA, to *L. tarentolae* supernatant or pellet followed by the assessment of SAP activity resulted in very modest effects on the supernatant activity (Table S1). These pulses had an only slightly larger effect on pellet acid phosphatase activity. The greatest effects on pellet activity were observed from the application of 250 µA (5% inhibition in activity as compared to control) and 400 µA (7% increase in activity compared to control). No apparent trends were observed in the supernatant or pellet groups, under these conditions.

The application of a 10,000 Hz, monophasic cathodic electric field, with amplitudes ranging from 100 to 500 µA, to *L. tarentolae* supernatant or pellet followed by the assessment of SAP activity resulted in different effects in both the supernatant and pellet activity that were statistically different from their controls (Table S2). The greatest effect in supernatant activity was observed from the 500 µA pulse (300% increase in activity when compared to control). There was an apparent non-linear trend in the activation of the supernatant activity from 300 to 500 µA, where the application of larger pulse amplitudes (current) leads to more activity. The greatest effect on pellet activity was also observed from the application of the 500 µA pulse (64% decrease in activity compared to control), however no apparent trends were observed in the pellet group, under these conditions.

The application of a 10,000 Hz symmetric biphasic electric fields (100 to 500 µA) to *L. tarentolae* supernatant or pellet, followed by the assessment of acid phosphatase activity resulted in very modest measurable effects in both the supernatant and pellet activity that were statistically different from their controls (Table S2). The greatest effect on supernatant activity was observed from the application of 200 µA (4% increase in activity as compared to control). The pellet activity was more influenced by these pulses. The greatest increase in pellet activity was observed from the application of the 150 µA pulse (7% increase in activity compared to control), and the greatest decrease in activity was observed from the application of the 500 µA pulse (11% decrease in activity when compared to control). No apparent trends were observed in the supernatant or pellet groups, under these conditions.

The application of a 10,000 Hz, monophasic anodic electric field fields (100 to 500 µA) to *L. tarentolae* supernatant or pellet, followed by the assessment of SAP activity resulted in very modest measurable effects in both the supernatant and pellet activity that were statistically different from their controls (Table S2). The pellet activity was both increased and decreased by these pulses. The greatest increase observed was from the application of 500 µA (5% increase in activity as compared to control). The greatest decrease in activity was observed from the application of 150 µA (9% decrease in activity when compared to control). No apparent trends were observed in the pellet group, under these conditions.

### 4.5. Effect of Electric Fields on SAP Secretion (Method 2)

To determine whether electric fields affected the secretion of SAPs, we also tested the effect of applying electric fields to whole cells prior to the fractionation to supernatant and pellet. All conditions tested produced only modest effects on supernatant activity (Method 2, Tables S3 and S4). The largest significant effect on pellet SAP activity was observed under the following conditions: (1) 50 Hz, symmetric biphasic pulses at 100 µA lead to a 36% decrease in pellet activity as compared to control; (2) 10, 000 Hz, monophasic cathodic pulses, 400 µA lead to an 11% increase in pellet activity compared to control; (3) 10,000 Hz, symmetric biphasic pulses, 200 µA lead to a 13% increase in activity compared to control; (4) 10,000 Hz, symmetric biphasic pulses, 500 µA lead to a 12% decrease in activity compared to control; and, (5) 10,000 Hz, monophasic anodic pulses, 250 µA lead to a 13% increase in activity when compared to control.

## 5. Discussion

### 5.1. Phenotypic and Viability Effects of Electric Fields on L. tarentolae Promastigotes

We have evaluated *L. tarentolae* cell viability (MTT assay), and their motility and clumping (microscopy). The electric fields that were tested in this work have no apparent effect on cell viability as compared to unexposed controls. We interpret this as likely being a specific effect rather than just due to general cell death. Thus, affecting either secretion of the enzymes or activity of the enzymes post secretion is of interest in and of itself and it may open up a completely new area for basic research into understanding the biochemistry of infection. 

However, the cells appeared to respond to electric fields by clumping or aggregation beneath the electrode in a manner that was differentially affected by the electric field frequency and polarity. We found that the application of symmetric biphasic and anodic electric fields caused cells not only to aggregate, but also to clump. Cell clumping may indicate cellular stress as cell membranes are modified and sticky cellular contents are extruded. In cell clumps, *L. tarentolae* may exhibit a behavior identified as quorum sensing. During quorum sensing, the cells restrict the expression of specific genes to the high cell densities at which the resulting phenotypes will be most beneficial to survival [24].

### 5.2. SAP Enzyme Kinetics and the Effect of Glycosylation

We have evaluated *L. tarentolae* promastigote SAP activity from log phase cells and found an apparent Michaelis-Menten type response to increasing substrate concentration. However, a Lineweaver-Burk transformation produced two different straight lines, starting on day three of culture. Thus, we concluded that there were two apparent enzymes detectable with different K_m_ or V_max_ values during the growth curve. These kinetic data correlate well with previously published genomics, indicating the existence of two separate enzymes [9]. While the genome of *L. tarentolae* has been sequenced [16], future functional studies need to be carried out to confirm enzyme identification.

The observation that SAP1 activity was detected on all eight days of the *L. tarentolae* growth curve suggests that this enzyme might play a role beyond that of aiding in survival of the parasite in the sand fly or aiding in alternative host infectivity. The observation that SAP2 activity was not detected until culture day three, in addition to its lower V_max_ on days three to six, followed by the increase in V_max_ upon culture senescence, suggests that SAP2 may be stored and released temporally. We speculate that these changes in V_max_ are due to changes in the amount of enzyme present, not actual changes in the kinetic constant, V_max_. We report that SAP1 consistently binds substrate less tightly (has a larger K_m_ value) than SAP2. Since this kinetic value is not dependent on enzyme concentration, the change in substrate binding by the two putative enzymes is of interest.

The importance of the carbohydrate moieties attached to *L. tarentolae* SAP1 or SAP2 enzyme was demonstrated with the incubation of the enzyme pool with PNGase F, followed by the assessment of SAP1 or SAP2 kinetic constants. The kinetic parameters of SAP1 and SAP2 were altered after PNGase F incubation (Tables S1 and S2). These results indicate that N-linked carbohydrates are functionally critical for either substrate binding, product formation, or to maintain enzyme solubility or structure. This result further supports the results of others [7] that there are two isoforms of SAP that are released by *L. tarentolae* into the culture medium, and that SAP1 and SAP2 may have different roles in successful parasite infections.

### 5.3. Electrical Stimulation Wave Form Comparisons

Overall, electric fields had the effect of increasing detectable SAP activity from *L. tarentolae* log phase enzyme pools. Of the 84 experiments done using method 1, varying Hertz, amperage, or waveform, 72 trials were significantly different relative to control (non-electrically) cells (Tables S1 and S2). Of these, thirty eight conditions significantly affected the supernatant relative to control whereas 34 conditions significantly affected pellets relative to control pellets. Thirty conditions affected both the supernatant and pellet fractions relative to controls. In contrast, using method 2 (Tables S3 and S4), only 45 of the 84 trials, varying Hertz, amperage, or waveform, resulted in significant differences relative to non-stimulated cells. Of the 45, only 19 conditions significantly affected the supernatant and 26 conditions affected the pellet responses relative to the control cells. Thus, both the total number of statistically significant results is larger when using method 1 (testing direct effects of electrical stimulation on enzyme activity), and the overall magnitude of the inhibitory effect, with the application of 10,000 Hz electric fields, is larger on *L. tarentolae* cell pellets when using method 1. The magnitude of the activation effect on *L. tarentolae* cell pellets, caused by the application of 10,000 Hz magnetic fields, is largest when using method 2.

## 6. Conclusions

### Potential for Synergistic Therapeutic Treatment for Leishmaniasis

Results from this work suggest that *Leishmania* secreted acid phosphatases, which were reported to be important in all species of *Leishmania* [12,25,26], remain as potential therapeutic targets for the treatment of leishmaniasis, and that treatment with electric fields may present a critical enhancement to available treatment options. This work correlates with the work by Giladi et al. [20] who indicated that 10 MHz, a much larger value of Hz than used in the current work, significantly inhibited bacterial growth *in vivo*. From our studies, which demonstrated the modulation in the enzyme activity while using electrical stimulation, either by directly affecting enzyme activity or by modulation of the release of the cell associated secreted acid phosphatase prematurely or too slowly, we speculate that this could affect the critical parasite-host interactions needed for successful infections. We suggest that parasite migration to a focal area by the application of electrical fields could increase the effectiveness of topical therapeutics, and thus, might be a useful combined therapy for the treatment of cutaneous leishmaniasis. The exposure to electric field pulses in this study were limited to 30 min. Thus, other exposure times should be tested to help determine the full therapeutic potential. One corollary that could be drawn from this study is that electric field therapy could be implemented through inexpensive, readily available electrical stimulator devices that could be applied to humans or domestic animals. In contrast to drug therapies, we anticipate that these devices could have fewer side effects, and they have a minimal risk of developing resistance to the therapy. Whether leishmaniasis treatment with a mild electric field followed by the application of a topical therapy is more effective than just the topical treatment alone remains to be determined in future studies. Clearly more work, such as tests using the amastigote for and/or in *vivo Leishmania* infective models, are worth considering.

## Supplementary Materials

The following are available online at http://www.mdpi.com/2076-0817/7/4/77/s1.
