# Supplementary material for: Effects of Specific Electric Field Stimulation on the Release and Activity of Secreted Acid Phosphatases from Leishmania tarentolae and Implications for Therapy
Source: Pathogens. 2018 Sep 27;7(4):77. doi: 10.3390/pathogens7040077 (PMC6313409; doi:10.3390/pathogens7040077)
Supplement: Supplementary file 1 [file pathogens-07-00077-s001.pdf]

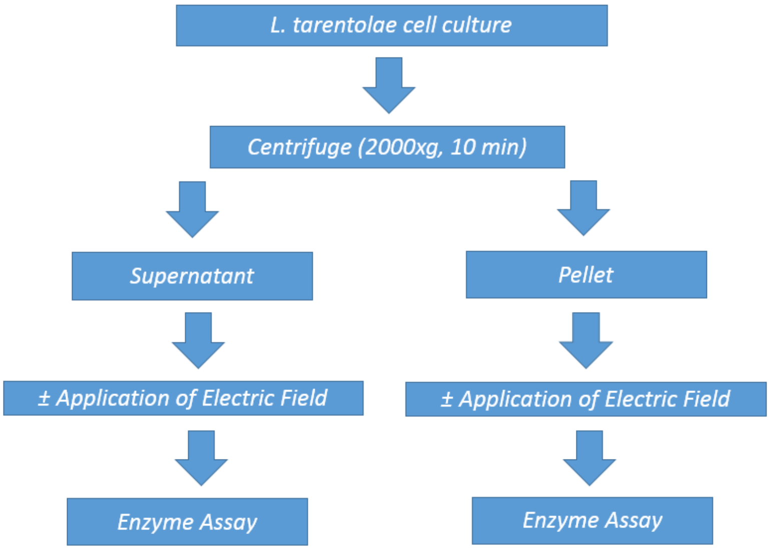

Supplemental Figure S1. Flow diagram of method 1. Thus, method 1 allows testing of direct effects of electric field on the previously secreted enzyme.

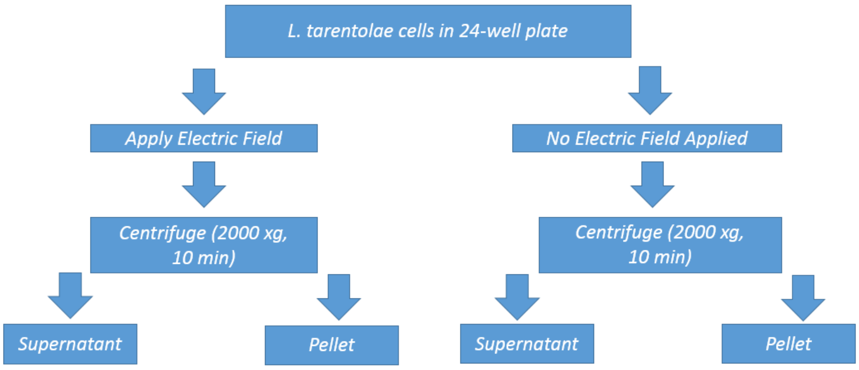

Supplemental Figure S2. Flow diagram of method 2 with enzyme assays performed on the subsequent supernatant and pellet fractions. Thus, method 2 allows assessment of the effect of electric field on secretion of acid phosphatase from cells.

Table S1. Effects of Electrical Stimulation, at 50 Hz Applied After Fractionation, on SAP Activity.

50 Hz Cathodic Monophasic				
**Current (μA)**	**Supernatant Percent Difference**		**Pellet Percent Difference**	
	**Mean ± SD**	**P-Value**	**Mean ± standard Deviation**	**P-Value**
100	5.22 ± 0.12	< 0.0001	−2.88 ± 0.34	< 0.0001
150	0.34 ± 0.11	0.0158	1.51 ± 0.16	0.0013
200	1.77 ± 0.11	< 0.0001	0.84 ± 0.28	0.0213
250	1.31 ± 0.29	0.0031	−1.76 ± 0.58	0.0062
300	−1.763 ± 0.006	0.0558	−1.13 ± 0.15	0.0029
400	−0.86 ± 0.11	0.0006	2.17 ± 0.83	0.0147
500	3.09 ± 0.28	< 0.0001	0.51 ± 0.44	0.2879
**50 Hz Symmetric Biphasic**				
**Current (μA)**	**Supernatant Percent Difference**		**Pellet Percent Difference**	
	**Mean ± standard Deviation**	**P-Value**	**Mean ± standard Deviation**	**P-Value**
100	−6.62 ± 0.24	0.4976	6.75 ± 0.22	0.0249
150	4.01 ± 0.39	0.0029	2.19 ± 0.61	0.0006
200	−1.60 ± 0.18	< 0.0001	−6.83 ± 0.92	0.0002
250	−0.241 ± 0.000	0.0011	−2.77 ± 0.60	< 0.0001
300	1.00 ± 0.34	0.0249	−1.41 ± 0.50	0.2051
400	0.979 ± 0.069	0.0039	−1.02 ± 0.34	< 0.0001
500	−1.34 ± 0.17	0.0061	0.47 ± 0.98	0.0721
**50 Hz Anodic Monophasic**				
**Current (μA)**	**Supernatant Percent Difference**		**Pellet Percent Difference**	
	**Mean ± standard Deviation**	**P-Value**	**Mean ± standard Deviation**	**P-Value**
100	−0.28 ± 0.59	0.0013	−1.15 ± 0.28	0.0019
150	0.52 ± 0.14	0.0001	1.68 ± 0.29	0.0377
200	−0.635 ± 0.068	0.0001	6.46 ± 0.80	0.7418
250	−0.67 ± 0.14	< 0.001	−5.496 ± 0.015	0.0151
300	−0.27 ± 0.13	0.0003	−1.33 ± 1.44	< 0.0001
400	−0.735 ± 0.066	< 0.0001	7.09 ± 0.31	0.0198
500	−0.54 ± 0.18	0.0003	3.12 ± 2.24	0.0009

Shown are the average percent difference from control ± SD for monophasic cathodic, symmetric biphasic, and monophasic anodic, at a frequency of 50 Hz, where fractionation occurred before electrical stimulation (Method 1). Statistical significance relative to control cells was determined by two-tailed Student's T-Tests. n = 3; $\alpha = 0.05$. Negative mean values indicate values lower than control (no electrical stimulation) cell values.

Table S2. Effects of Electrical Stimulation, at 10,000 Hz Applied After Fractionation, on SAP Activity.

**10,000 Hz Cathodic Monophasic**				
**Current (μA)**	**Supernatant Percent Difference**		**Pellet Percent Difference**	
	**Mean ± standard Deviation**	**P-Value**	**Mean ± standard Deviation**	**P-Value**
100	2.38 ± 0.15	0.0013	−2.90 ± 0.22	0.0019
150	10.18 ± 0.18	< 0.0001	−0.12 ± 0.58	0.0377
200	16.47 ± 0.18	< 0.0001	−3.431 ± 0.008	0.7418
250	6.77 ± 0.21	< 0.0001	−10.83 ± 0.61	0.0151
300	15.433 ± 0.084	0.0003	−4.506 ± 0.010	< 0.0001
400	22.62 ± 0.22	< 0.0001	−4.51 ± 0.43	0.0198
500	307.56 ± 0.18	0.0003	−64.43 ± 2.15	0.0009
**10,000 Hz Symmetric Biphasic**				
**Current (μA)**	**Supernatant Percent Difference**		**Pellet Percent Difference**	
	**Mean ± standard Deviation**	**P-Value**	**Mean ± standard Deviation**	**P-Value**
100	3.84 ± 0.16	0.0006	−9.17 ± 0.54	0.0014
150	3.18 ± 0.28	0.0003	7.30 ± 0.60	0.0340
200	4.42 ± 0.28	0.0025	−6.99 ± 0.23	0.0022
250	1.707 ± 0.083	0.0003	−4.97 ± 0.29	0.0109
300	1.495 ± 0.002	0.0013	−8.62 ± 0.30	0.0005
400	−0.354 ± 0.077	0.0010	−5.723 ± 0.021	0.0003
500	1.20 ± 0.13	0.0007	−11.91 ± 0.23	0.0011
**10,000 Hz Anodic Monophasic**				
**Current (μA)**	**Supernatant Percent Difference**		**Pellet Percent Difference**	
	**Mean ± standard Deviation**	**P-Value**	**Mean ± standard Deviation**	**P-Value**
100	10.42 ± 0.21	< 0.0001	−1.59 ± 0.16	0.0021
150	11.52 ± 0.11	< 0.0001	−9.65 ± 1.17	0.0003
200	11.10 ± 0.26	< 0.0001	−4.79 ± 0.37	0.0061
250	16.009 ± 0.066	< 0.0001	15.99 ± 0.32	0.1994
300	16.90 ± 0.41	< 0.0001	−2.50 ± 0.14	0.0022
400	16.209 ± 0.031	< 0.0001	−1.88 ± 0.98	0.0002
500	17.697 ± 0.098	< 0.0001	4.90 ± 0.90	< 0.0001

Shown are the average percent difference from control ± SD for monophasic cathodic, symmetric biphasic, and monophasic anodic, at a frequency of 10,000 Hz, where fractionation occurred before electrical stimulation (Method 1). Statistical significance relative to control cells was determined by two-tailed Student's T-Tests. $n = 3$; $\alpha = 0.05$. Negative mean values indicate values lower than control (no electrical stimulation) cell values.

Table S3. Effects of Electrical Stimulation, at 50 Hz Applied Before Fractionation, on SAP Activity and Secretion.

**50 Hz Cathodic Monophasic**				
**Current (μA)**	**Supernatant Percent Difference**		**Pellet Percent Difference**	
	**Mean ± standard Deviation**	**P-Value**	**Mean ± standard Deviation**	**P-Value**
100	5.59 ± 0.30	0.0004	−1.531 ± 0.096	0.0065
150	2.17 ± 0.26	< 0.0001	0.15 ± 0.26	0.3739
200	−0.87 ± 0.71	0.0894	−5.21 ± 0.46	0.0002
250	0.34 ± 0.34	0.1012	1.29 ± 0.32	0.0080
300	−2.25 ± 0.33	0.0006	2.87 ± 0.57	0.0018
400	1.347 ± 0.095	0.0075	6.51 ± 0.24	< 0.0001
500	−1.11 ± 0.16	0.0003	1.21 ± 0.16	0.0139
**50 Hz Symmetric Biphasic**				
**Current (μA)**	**Supernatant Percent Difference**		**Pellet Percent Difference**	
	**Mean ± standard Deviation**	**P-Value**	**Mean ± standard Deviation**	**P-Value**
100	−3.26 ± 1.99	0.1330	−36.20 ± 0.70	< 0.0001
150	1.30 ± 1.11	0.0862	2.20 ± 0.61	0.1708
200	0.48 ± 0.73	0.5593	−8.38 ± 3.50	0.0669
250	−0.57 ± 0.75	0.4121	−2.59 ± 1.42	0.1700
300	1.10 ± 0.69	0.2035	2.48 ± 3.60	0.3545
400	0.05 ± 0.66	0.984	2.51 ± 1.67	0.1145
500	0.57 ± 0.79	0.7004	−2.46 ± 0.78	0.0335
**50 Hz Anodic Monophasic**				
**Current (μA)**	**Supernatant Percent Difference**		**Pellet Percent Difference**	
	**Mean ± standard Deviation**	**P-Value**	**Mean ± standard Deviation**	**P-Value**
100	0.13 ± 3.17	0.9353	−4.255 ± 0.000	< 0.0001
150	0.003 ± 0.976	1.0000	−2.151 ± 0.005	0.0003
200	0.38 ± 0.55	0.3739	0.49 ± 0.12	0.0572
250	1.29 ± 0.56	0.0474	0.21 ± 0.41	0.6240
300	0.00 ± 0.00	1.0000	2.34 ± 0.13	0.0015
400	4.05 ± 2.35	0.0406	1.64 ± 0.21	0.0023
500	−1.68 ± 4.52	0.4981	1.74 ± 0.24	0.0002

Shown are the average percent difference from control ± SD for monophasic cathodic, symmetric biphasic, and monophasic anodic, at a frequency of 50 Hz, where electrical stimulation occurred before fractionation (Method 2). Statistical significance relative to control cells was determined by two-tailed Student's T-Tests. $n = 3$; $\alpha = 0.05$. Negative mean values indicate values lower than control (no electrical stimulation) cell values.

Table S4. Effects of Electrical Stimulation, at 10,000 Hz Applied Before Fractionation, on SAP Activity and Secretion

**10,000 Hz Cathodic Monophasic**				
**Current (μA)**	**Supernatant Percent Difference**		**Pellet Percent Difference**	
	**Mean ± standard Deviation**	**P-Value**	**Mean ± standard Deviation**	**P-Value**
100	−0.76 ± 0.40	0.0916	−2.43 ± 0.40	0.0002
150	−0.86 ± 0.38	0.0434	2.79 ± 0.40	0.0003
200	0.60 ± 0.30	0.1583	−5.01 ± 0.21	< 0.0001
250	−0.54 ± 0.31	0.0295	−1.873 ± 0.000	< 0.0001
300	1.81 ± 0.17	0.0002	−1.93 ± 0.54	0.0129
400	1.42 ± 0.17	< 0.0001	11.83 ± 0.25	< 0.0001
500	1.23 ± 0.58	0.0291	−2.88 ± 0.46	0.0022
**10,000 Hz Symmetric Biphasic**				
**Current (μA)**	**Supernatant Percent Difference**		**Pellet Percent Difference**	
	**Mean ± standard Deviation**	**P-Value**	**Mean ± standard Deviation**	**P-Value**
100	−1.06 ± 0.74	0.5299	54.47 ± 1.26	0.1240
150	0.64 ± 0.31	0.0314	2.01 ± 1.39	0.1000
200	0.00 ± 0.15	1.0000	13.096 ± 0.078	< 0.0001
250	−0.74 ± 0.51	0.0816	1.17 ± 0.59	0.0550
300	0.74 ± 0.26	0.0179	4.40 ± 0.34	< 0.0001
400	−0.19 ± 0.73	0.6433	−0.35 ± 0.80	0.5614
500	−1.26 ± 0.28	0.0032	−12.21 ± 0.89	0.0000
**10,000 Hz Anodic Monophasic**				
**Current (μA)**	**Supernatant Percent Difference**		**Pellet Percent Difference**	
	**Mean ± standard Deviation**	**P-Value**	**Mean ± standard Deviation**	**P-Value**
100	−0.86 ± 1.48	0.2030	3.49 ± 4.22	0.2227
150	0.248 ± 0.086	0.2520	4.48 ± 0.52	0.0001
200	−5.525 ± 0.075	< 0.0001	3.89 ± 0.30	< 0.0001
250	1.47 ± 0.14	0.0020	13.79 ± 2.88	0.0008
300	−0.43 ± 0.14	0.0030	−4.47 ± 0.55	0.0002
400	1.55 ± 0.51	0.0040	−2.32 ± 0.46	0.0036
500	1.70 ± 0.86	0.0640	1.843 ± 0.008	0.0080

Shown are the average percent difference from control ± SD for monophasic cathodic, symmetric biphasic, and monophasic anodic, at a frequency of 10,000 Hz, where electrical stimulation occurred before fractionation (Method 2). Statistical significance relative to control cells was determined by two-tailed Student's T-Tests. n = 3; $\alpha = 0.05$. Negative mean values indicate values lower than control (no electrical stimulation) cell values.
